# Lifelong exercise, but not short‐term high‐intensity interval training, increases GDF11, a marker of successful aging: a preliminary investigation

**DOI:** 10.14814/phy2.13343

**Published:** 2017-07-12

**Authors:** Bradley T. Elliott, Peter Herbert, Nicholas Sculthorpe, Fergal M. Grace, Daniel Stratton, Lawrence D. Hayes

**Affiliations:** ^1^ Department of Biomedical Sciences University of Westminster London United Kingdom; ^2^ School of Sport, Health and Outdoor Education Trinity Saint David University of Wales Cardiff United Kingdom; ^3^ Institute of Clinical Exercise and Health Science University of the West of Scotland Paisley United Kingdom; ^4^ Faculty of Health Federation University Ballarat Victoria Australia; ^5^ Cellular and Molecular Immunology Research Center London Metropolitan University London United Kingdom; ^6^ Active Ageing Research Group Department of Medical and Sport Sciences University of Cumbria Carlisle United Kingdom

**Keywords:** Aging, exercise, follistatin, GDF11, HIIT, myostatin

## Abstract

Lifelong exercise is associated with regulation of skeletal mass and function, reductions in frailty, and successful aging. Yet, the influence of exercise on myostatin and myostatin‐interacting factors is relatively under examined in older males. Therefore, we investigated whether serum total myostatin, free myostatin, follistatin, and growth and differentiation factor 11 (GDF11) were altered following high‐intensity interval training (HIIT) in a group of 13 lifelong sedentary (SED; 64 [6] years) and 11 lifelong exercising (LEX; 62 [6] years) older males. SED follistatin was moderately greater than LEX pre‐HIIT (Cohen's *d* = 0.66), and was largely greater post‐HIIT (Cohen's *d* = 1.22). The HIIT‐induced increase in follistatin was large in SED (Cohen's *d* = 0.82) and absent in LEX (Cohen's *d* = 0.03). GDF11 was higher in LEX pre‐HIIT (Cohen's *d* = 0.49) and post‐HIIT (Cohen's *d* = 0.63) compared to SED. HIIT resulted in no change to GDF11 in LEX or SED (Cohen's *d* = 0.00–0.03). Peak power output and GDF11 were correlated (*r* = 0.603), independent of grouping. Differences in GDF11 with lifelong exercise training, paired with the correlation between GDF11 and peak power output, suggested that GDF11 may be a relevant myostatin‐interacting peptide to successful aging in humans, and strategies to maintain this need to be further explored.

## Introduction

Myostatin (originally growth and differentiation factor 8 [GDF8]) is a procatabolic, antianabolic peptide hormone that is a central regulator of skeletal muscle mass (Elliott et al. 2012). Secreted by the skeletal muscle, myostatin is found in an active unbound (free) form, or bound to its own propeptide, or separate peptides such as follistatin, or follistatin‐related gene (FLRG; Hill et al. 2002; Amthor et al. 2004; Gilson et al. 2009), each inhibiting its biological function. Myostatin has both paracrine and endocrine effects (Zimmers et al. 2002), although it is the endocrine function which appears key for regulation of muscle mass, due to an observed inverse correlation with muscle mass in humans (Gonzalez‐Cadavid et al. 1998). Moreover, inhibition of this endocrine function results in muscle hypertrophy in mice (Whittemore et al. 2003).

Aging is associated with a progressive loss of muscle mass and associated function (Metter et al. 2002). The rate of loss of muscle mass and function with aging is noted to differ between individuals, which gave rise to “usual” and “successful” aging hypothesis (Rowe and Kahn 1987). A more recent definition of successful aging being “optimisation of life expectancy while minimising physical and mental deterioration and disability” (Bowling and Dieppe 2005), a trait that is often seen in lifelong masters athletes (Pollock et al. 2015). While the role of myostatin in regulation of muscle mass is well described, there are few data and no prospective studies to contextualize the influence of myostatin within the “cycle of frailty” that precedes sarcopenia. From the few cross‐sectional studies, one observed ~50% higher plasma myostatin in older sedentary (SED; ~63–75 years of age) compared with younger healthy (~20–35 years of age) men (Yarasheski et al. 2002). However, this was not replicated in a study of men aged ~22, ~69, and ~76 years of age, regardless of sarcopenic severity (Ratkevicius et al. 2011). Recently, we have observed an inverse association between age and plasma myostatin in a large group (*n* = 88) of healthy individuals aged 18–72 years of age (Elliott et al. 2016). Considering the current incomplete understanding concerning the role of myostatin and myostatin‐interacting peptides in the aging process, the pool of evidence needs to be extended.

Growth and differentiation factor 11 (GDF11) is a peptide with similar sequence homology as myostatin, and it is possible that both peptides share similar signaling pathways and biological influence within the skeletal muscle. Unlike myostatin, however, the expression of GDF11 is not limited to skeletal muscle tissue (Lee and McPherron 1999; Walker et al. 2016). There also appears to be an indicated role for GDF11 in the aging process; higher circulating GDF11 in middle‐aged mice has been positively associated with longevity and exposure of aged mice to a youthful systemic environment led to restoration of skeletal muscle and hepatic cellular function (Zhou et al. 2016). Similarly, the aging muscle phenotype is partially offset by provision of recombinant GDF11, as demonstrated by increased grip strength and running endurance in mice (Sinha et al. 2014).

While it remains to be seen whether these findings can be consistently replicated, or indeed translated to the human model of aging, only a small number of studies that have examined the effects of exercise training on serum myostatin and associated mRNA expression, while GDF11 remains unexamined in the human exercise model. Indeed, 2–3 months' resistance training in healthy young individuals resulted in increased muscle mass and decreased muscle mRNA and serum myostatin (Roth et al. 2003; Walker et al. 2004). To the best of these authors' knowledge, no reports on the effect of exercise (in any form) on GDF11 expression currently exist.

Recently, high‐intensity interval training (HIIT) has received much attention due to its physiological and sociological benefits. Indeed, HIIT is noted to be more enjoyable than traditional, continuous training (Thum et al. 2017), has higher compliance in patient populations than continuous training (Shiraev and Barclay 2012), and is noted to have equal or improved clinical outcomes in a number of aging‐related cardiovascular or metabolic disorders (Ramos et al. 2015; Cassidy et al. 2016). While not optimized for muscle hypertrophy, HIIT improves myofibrillar protein synthesis (Bell et al. 2015), muscle power (Sculthorpe et al. 2017), and fat‐free mass (FFM) (Herbert et al. 2017) in older males.

Therefore, in order to progress our understanding of the biological relationship between myostatin and myostatin‐interacting peptides with aging and exercise, the aim of this preliminary study was twofold: (1) to compare resting levels of plasma myostatin and myostatin‐interacting peptides between lifelong SED and a positive control group of lifelong exercising (LEX) aging men, and (2) to examine the influence of 6 weeks' HIIT on plasma myostatin and myostatin‐interacting peptides in SED and LEX. We hypothesized that on enrollment to the study SED would exhibit higher myostatin, follistatin, free myostatin, and lower GDF11. We further hypothesized that 6 weeks' HIIT would decrease plasma myostatin, follistatin, and free myostatin in SED, and increase GDF11.

## Methods

### Participants

Participants provided written informed consent prior to enrollment to a larger study (Knowles et al. 2015; Hayes et al. 2017; Herbert et al. 2017), which was approved by the University of the West of Scotland Ethics Committee (Reference: UEC16_042012/Herbert). Participants were familiarized with experimental procedures and approval to exercise was given by their general practitioner. Subsequently, a subgroup of 24 males was analyzed for this pilot investigation. Thirteen males participated in the SED group, while 11 males participated in the LEX group (Table 1). Participants in the SED group did not take part in any formal exercise training and had not done so for >30 years. The LEX group were active exercisers and had been so for the previous >30 years. They consisted primarily of current masters competitors in sports including water polo, triathlon, sprint cycling, road cycling, and distance running. For 6 weeks prior to commencing HIIT training, LEX recorded their normal weekly exercise, which included type, frequency, intensity (recorded by heart rate telemetry), and duration of training. Time spent in low to medium intensity (<65% heart rate reserve [HRR]), and high‐intensity (>65% HRR) training totaled 214 ± 131 and 67 ± 52 min/week, respectively. Group selection was affirmed by differences in aerobic conditioning (peak oxygen uptake; *V*O_2peak_) between groups (Table 1). Participants were tested pre‐ and post‐HIIT at the same time of day, 7 weeks apart. Order of measurements was blood sampling, body composition, peak power assessment, and determination of *V*O_2peak_.

**Table 1:** Participant anthropometric and performance parameters on enrollment to the investigation in lifelong SED, and LEX, older males

	SED (*n* = 13)	LEX (*n* = 11)
Age, years	64 (6)	62 (6)
Stature, cm	174 (6)	174 (6)
Body mass, kg	91 (19)	80 (12)
Body fat, %	24 (16)	16 (6)
FFM, kg	66 (6)	66 (7)
Peak oxygen uptake, mL/kg per min	28 (6)	40 (7)^a^
Peak power output, W	663 (147)	831 (221)^a^
Peak power output, W/kg FFM	10 (2)	12 (2)^a^

Data presented as mean (SD). FFM, fat‐free mass; SED, sedentary; LEX, lifelong exercising.

a Denotes significantly different than SED (*P* < 0.05).

### Blood draws and analysis

Participants arrived at the exercise physiology laboratory between 0700 and 0900 h, following an overnight fast and having abstained from strenuous exercise for a minimum of 48 h. Participants were reminded to maintain standardized conditions prior to each assessment point which included arriving in a hydrated state having abstained from caffeine and alcohol consumption for 36 h. Following 20 min supine rest, blood was sampled from the nondominant arm using the standard venipuncture method into sterile serum separator vacutainer tubes (Becton Dickinson, Rutherford, NJ) that were kept at room temperature in the dark, for 30 min, to allow for clotting, after which samples were centrifuged at 1100*g* at 4°C for 15 min. Serum was then extracted, aliquoted, and stored at −80°C until subsequent analysis. Blood samples were collected at the same time of day for each participant to control for biological variation and minimize interparticipant analytical variation.

Concentrations of serum myostatin protein (both total and free fractions) were quantified by enzyme‐linked immunosorbent assay (ELISA; DGDF80; R&D Systems, UK). Briefly, aliquots of serum were brought to room temperature, before 100 *μ*L of plasma was diluted with 1:4 diluent buffer (free myostatin) or activated with 50 *μ*L HCl (6 mol, 10 min at room temperature) for removal of myostatin binding proteins, before neutralization (50 *μ*L of NaOH 6 mol + 1.2 mol 4‐(2‐hydroxyethyl)‐1‐piperazineethanesulfonic acid) and dilution with provided diluent buffer (200 *μ*L) to produce a final 1:4 dilution. Recombinant myostatin was used as a standard (33.3–2000 pg/mL). Concentrations of serum follistatin (DFN00; R&D Systems, Abingdon, UK) and serum GDF11 (DY1958; R&D Systems) were quantified by ELISA, per manufacturer's instructions. Recombinant follistatin (250–16,000 pg/mL) and GDF11 (15.6–1000 pg/mL) were as standards. Plates were read spectrophotometrically at 450 nm and blanked to 570 nm (VersaMax; Molecular Devices, CA). Coefficient of variability of standards and samples were 7% and 6%, 6% and 4%, and 4% and 8%, for myostatin follistatin, and GDF11, respectively.

### Body composition and performance measures

Height was measured to the nearest 0.1 cm using a stadiometer (Seca, Birmingham, UK), and a multifrequency bioelectrical impedance analyzer (BIA; Tanita MC‐180MA Body Composition Analyzer, Tanita, Chicago, IL) was used to determine body mass and body composition as described elsewhere (Hayes et al. 2013b). Participant peak power output was assessed using the Herbert 6‐sec cycle test (Herbert et al. 2015b) and participants' individual values were used to calculate the resistance (40% peak power output) during HIIT. *V*O_2peak_ was determined by indirect calorimetry as previously described (Knowles et al. 2015).

### Exercise training

HIIT sessions were performed once every 5 days for 6 weeks (nine sessions in total) as previously described (Knowles et al. 2015; Hayes et al. 2017; Herbert et al. 2017). Rationale for this program is provided by our previous work which identified that 5 days of recovery was required for recovery of peak power output in aging men (Herbert et al. 2015a). Each session consisted of 6 × 30 sec sprints at 40% predefined peak power output interspersed with 3 min active recovery on a cycle ergometer (Wattbike Ltd., Nottingham, UK). Sessions were conducted in groups of between four and six participants and were the sole exercise performed by both groups during this time.

### Statistical analysis

Following confirmation of parametricity by a Shapiro–Wilk test of normality and Levene's test for homogeneity of variance, a mixed (between group [SED, LEX] × within individual time [pre‐HIIT and post‐HIIT]) repeated measures analysis of variance (ANOVA) was used for differences in groups and time points with Bonferroni post‐hoc. Nonparametric data were examined by Fishers exact test, with correction for multiple comparisons by Bonferroni's method. Alpha level was set a priori at *P* < 0.05, and effect size for paired comparisons is reported as Cohen's *d* throughout, interpreted as trivial (<0.2), small (≥0.2), moderate (≥0.5), and large (≥0.8). Parametric datasets are summarized in text as mean (SD), while nonparametric are given as median (upper − lower quartile). Figures are presented as grouped dot plots, as recommended by Drummond and Vowler (2012).

## Results

Pre‐HIIT, SED individuals were heavier (*P* = 0.131, Cohen's *d* = 0.66) with a greater body fat percentage (*P* = 0.120, Cohen's *d* = 0.66) than LEX. SED had a lower *V*O_2peak_ (*P* < 0.001, Cohen's *d* = 2.00), absolute peak power output (*P* = 0.036, Cohen's *d* = 0.90), and relative peak power output (a surrogate for muscle quality; *P* = 0.020, Cohen's *d* = 1.08) than LEX (Table 1).

**Figure 1:**
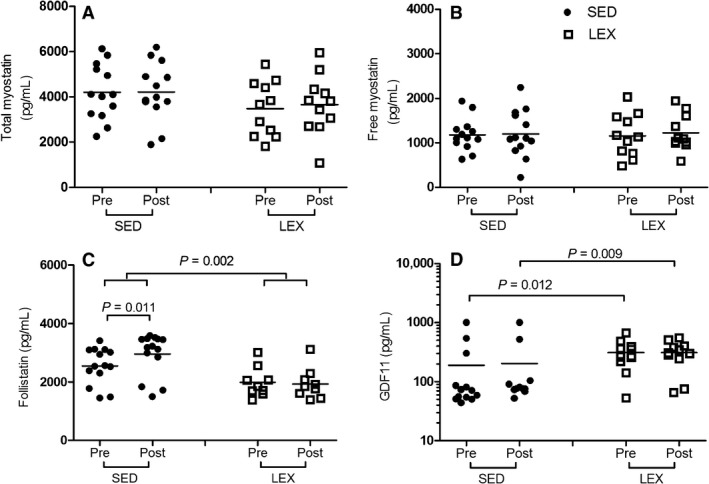
Effect of HIIT on growth factor family members in SED (*N* = 13) and LEX (*N* = 11) individuals. Concentration of (A) total myostatin, (B) free myostatin, (C) follistatin and (D) GDF11 pre‐ and post‐HIIT. Note (D) is expressed on a logarithmic *y* axis. HIIT, high‐intensity interval training; LEX, lifelong exercising; SED, sedentary; GDF11, growth and differentiation factor 11.

There was no group × time interaction for total myostatin protein (*P* = 0.750), nor was there an effect of group (*P* = 0.081) or time (*P* = 0.701). However, large effect sizes were noted between SED and LEX total myostatin both pre‐HITT (4217 [317] pg/mL and 3394 [391] pg/mL in SED and LEX, respectively; Cohen's *d* = 2.06; Fig. 1A) and post‐HIIT (4163 [337] pg/mL and 3678 [438] pg/mL in SED and LEX, respectively; Cohen's *d* = 1.24). Following HIIT, SED experienced only trivial increases in total myostatin (Cohen's *d* = 0.17) while LEX moderately increased total myostatin (Cohen's *d* = 0.68).

In a similar manner to total myostatin, there was no group × time interaction for free myostatin protein (*P* = 0.790), nor and effect of group (0.996) or time (*P* = 0.601). No notable effect size changes were observed for free myostatin pre‐HIIT (1182.0 [372.2] pg/mL and 1159.3 [418.1] pg/mL in SED and LEX, respectively; Cohen's *d* = 0.06; Fig. 1B) or post‐HITT (1203.3 [533.3] pg/mL and 1224.5 [404.1] pg/mL in SED and LEX, respectively; Cohen's *d* = 0.05). Moreover, neither SED (Cohen's *d* = 0.05) nor LEX (Cohen's *d* = 0.16) had any more than a trivial effect on free myostatin from pre‐ to post‐HIIT.

There was a significant main effect of group (*P* = 0.002), but not time (*P* = 0.171), or a group × time interaction (*P* = 0.561) for serum follistatin. SED follistatin was greater than LEX follistatin pre‐HIIT (2508 [628] pg/mL and 2102 [598] pg/mL in SED and LEX, respectively; *P* = 0.132, Cohen's *d* = 0.66). SED follistatin was also greater than LEX follistatin post‐HIIT (3043 [676] pg/mL and 2126 [809] pg/mL in SED and LEX, respectively; *P* < 0.001, Cohen's *d* = 1.22). The HIIT‐induced increase in follistatin was large in SED (*P* = 0.011, Cohen's *d* = 0.82), while LEX experienced no change (*P* = 0.443, Cohen's *d* = 0.03).

GDF11 data were examined by Fishers exact test, and presented as median (upper − lower quartile). GDF11 was higher in LEX pre‐HIIT (*P* = 0.012, Cohen's *d* = 0.49), and post‐HIIT (*P* = 0.009, Cohen's *d* = 0.63) compared to SED. HIIT resulted in no change to GDF11 in SED (70.7 [52.6–193.1], 77.1 [73.1–104.3] pg/mL pre‐ and post‐HIIT, respectively; *P* = 0.74, Cohen's *d* = 0.03) or LEX (272.7 [219.2–387.2], 305.0 [243.8–399.4] pg/mL pre‐ and post‐HIIT, respectively; *P* = 0.72, Cohen's *d* = 0.00).

**Figure 2:**
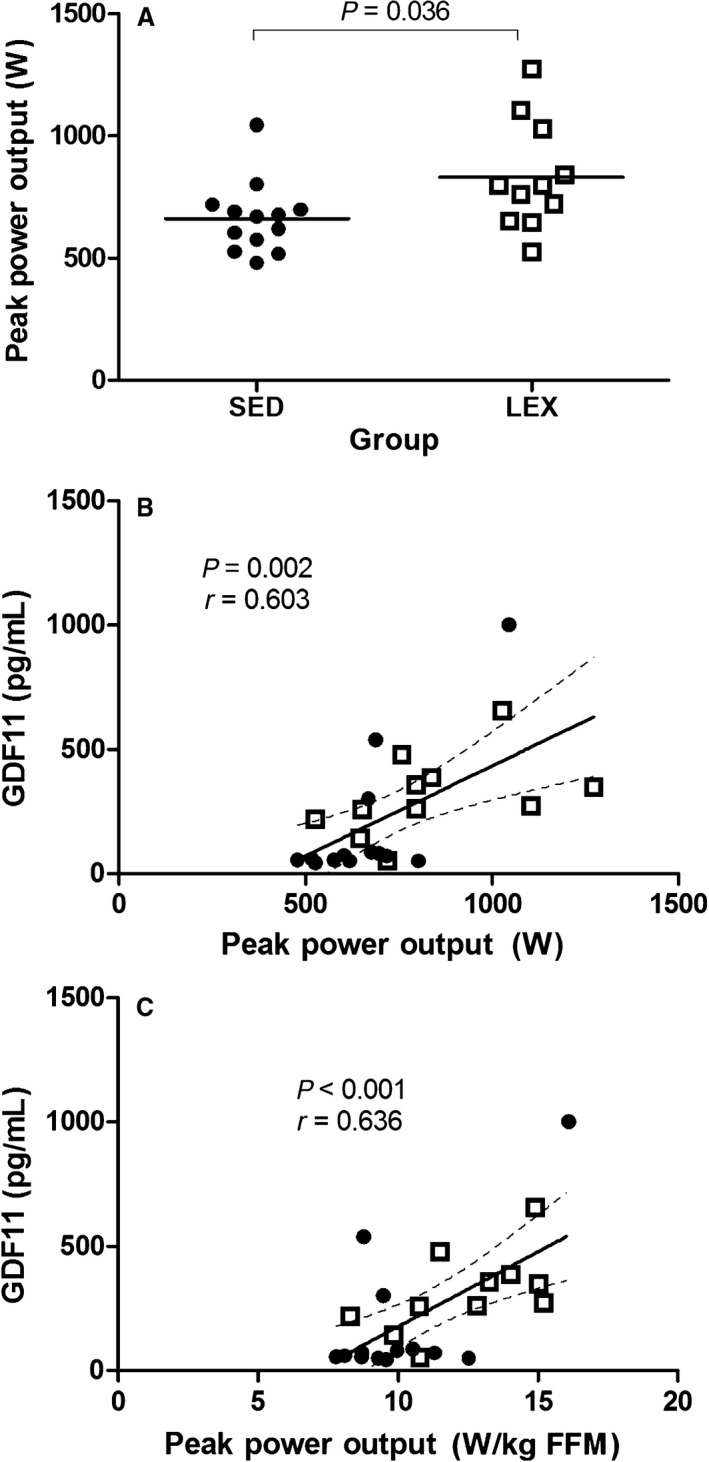
Correlations between myostatin‐interacting factors and peak power output (W). (A) Peak power output (W) by group (SED, LEX). Horizontal solid lines indicate group mean. (B) GDF11 (pg/mL) as a function of peak power output (W). (C) GDF11 (pg/mL) as a function of peak power output (W/kg FFM). Dashed line indicates 95% confidence intervals. Closed circles are SED (*N* = 13), open squares are LEX (*N* = 11). LEX, lifelong exercising; SED, sedentary; GDF11, growth and differentiation factor 11.

As we have previously reported in a larger cohort (Hayes et al. 2013a), peak power output was higher in LEX individuals relative to SED (*P* = 0.036, Fig. 2A). There was no correlation between peak power output and total myostatin (*P* = 0.196, *r* = −0.273), free myostatin (*P* = 0.812, *r* = 0.051), or follistatin (*P* = 0.569, *r* = −0.113). However, strong positive correlations were observed between GDF11 and both absolute peak power output (*P* = 0.002, *r* = 0.603; Fig. 2B) and relative peak power output (*P* < 0.001, *r* = 0.636; Fig. 2C).

## Discussion

The main finding of this preliminary study was that SED presented greater concentrations of serum total myostatin and follistatin, and lower concentrations of GDF11, compared with LEX pre‐HIIT. Serum follistatin alone responded significantly to HIIT but was confined to the SED group. A notable and novel finding from this study is the observed association between peak power output and GDF11, which has not been previously demonstrated in the human. These data provide preliminary evidence that the role of GDF11 in healthy aging observed in mice is maintained in humans.

With regard to healthy aging, our finding that LEX displayed significantly higher GDF11 than SED at baseline is novel and noteworthy. It has been noted that older mice treated with plasma from younger mice show a younger phenotype (Horrington et al. 1960; Lunsford et al. 1963), which has since been partially attributed to GDF11 differences in older and younger mice. In mice, midlife GDF11 is predictive of longevity (Zhou et al. 2016). Aged mice show a typical “older muscle” phenotype which results in lower muscle volume, endurance, and grip strength relative to young mice. Moreover, treatment with recombinant GDF11 returned grip strength to near young levels, and improved running endurance performance (Sinha et al. 2014). However, it has also been noted that GDF11 may inhibit myoblast differentiation into mature myotubes in a myostatin‐like manner (Egerman et al. 2015), perhaps unsurprising, as the myostatin and GDF11 peptide share ~90% homogeneity. It should be further noted that Egerman et al. (2015) used in vitro doses of 10–100 ng/mL, while both their data, and our data reported here, suggests circulating GDF11 in older males is 100–1000 pg/mL, an order of magnitude lower in concentration, possibly explaining the disparity of these findings.

This argument that GDF11 concentration plays a role in successful aging is supported by two separate findings we report here. First, we note GDF11 is significantly higher in LEX than SED, with some overlap between these groups. Further, we note a significant moderate positive correlation between peak power output (both absolute power and relative to FFM) and GDF11, independent of grouping. While our data does not allow us to suggest causality, it is exciting to note this correlative relationship. To the best of our knowledge, this is the first dataset linking successful aging and improved muscle function in the human with GDF11, and directly links our findings with those of Sinha et al. (2014), that exogenous GDF11 protects older mice against aging‐ and sedentarism‐associated frailty. It is thus tempting to suggest GDF11 plays a similar role in aging humans, and this hypothesis needs to be further explored with experimental approaches to increase GDF11 expression in humans.

Circulating myostatin is noted to correlate with lean muscle mass across both healthy and cachexic individuals (Gonzalez‐Cadavid et al. 1998). As SED and LEX presented with different body composition at baseline, the moderately lower concentrations of total myostatin in LEX at baseline is understandable. While others have reported decreases in plasma myostatin and gains in muscle mass following resistance exercise (Walker et al. 2004; Saremi et al. 2010), limited research regarding interaction between HIIT and myostatin exists. Pugh et al. (2015) reported reduced muscular myostatin mRNA in healthy individuals 2 and 6 h following a single bout of HIIT (although a different protocol to that employed herein), yet we are the first group to report chronic changes to resting serum myostatin following HIIT. The aim of HIIT is not primarily to build muscle mass, so while our HIIT protocol did not significantly alter serum total or free myostatin, expectations of an alteration in this peptide may have been ambitious in the absence of muscle mass alteration.

While our findings concerning GDF11 are noteworthy, we acknowledge certain limitations of the present investigation. While we attribute differences in GDF11 to lifelong activity differences, we acknowledge that we cannot separate how much exercise was required to produce these observed differences. The addition of a moderately active group (meeting physical activity guidelines), would allow for comparison of multiple exercise habits, rather than the two extremes presented here. Moreover, our lack of inactive control group (no HIIT) and relatively small sample size may limit interpretations. The present investigation formed part of a larger research study with other primary outcome variables (Grace et al. 2015; Knowles et al. 2015; Herbert et al. 2017), and therefore only a subset of participants were analyzed. As such, our results remain preliminary until the influence of exercise habits on serum GDF11 is investigated with either a large‐scale randomized control trial (RCT) or prospective observational trial.

To date, much attention has been placed on myostatin itself, with alterations in myostatin expression resulting in significant and striking alterations in muscle mass in animal models (Kambadur et al. 1997; Mosher et al. 2007). However, here, we show that total myostatin only moderately differs in a model of successful aging, suggesting the role of myostatin may not be as important in successful aging as other factors reported here. Instead, greater focus may need to be placed on these myostatin‐interacting factors, as we showed follistatin was lower, and GDF11 was higher in our LEX model of successful aging. Further, the correlation between GDF11 and muscle quality is exciting, and may suggest a protective role of GDF11 against aging‐associated muscular frailty in the human.

## Conflict of Interest

None declared.
